# Problematic Internet Usage and Self-Esteem in Chinese Undergraduate Students: The Mediation Effects of Individual Affect and Relationship Satisfaction

**DOI:** 10.3390/ijerph18136949

**Published:** 2021-06-29

**Authors:** Guang Zeng, Lijin Zhang, Sai-fu Fung, Jingwen Li, Yi-Man Liu, Zi-Ke Xiong, Zhi-Quan Jiang, Fang-Fang Zhu, Zhen-Ting Chen, Si-Ding Luo, Ping Yu, Qian Huang

**Affiliations:** 1 Department of Psychology, Sun Yat-sen University, Guangdong 510275, China; zengg6@mail.sysu.edu.cn (G.Z.); zhanglj37@mail2.sysu.edu.cn (L.Z.); 2 Department of Social and Behavioural Sciences, City University of Hong Kong, Hong Kong, China; 3 Department of Psychology, Tsinghua University, Beijing 100084, China; lijw921012@163.com; 4 School of Economics, Finance and Marketing, Royal Melbourne Institute of Technology, Melbourne, VIC 3001, Australia; lym.scg.cn@gmail.com; 5 Guangzhou Huashang College, University Administrative Office, Guangdong 511300, China; xzk_scg_cn@163.com (Z.-K.X.); jzq_edu_cn@163.com (Z.-Q.J.); 6 School of Economics and Trade, Guangzhou Huashang College, Guangdong 511300, China; tjx_jr@163.com; 7 School of Data Science, Guangzhou Huashang College, Guangdong 511300, China; z.t_chen@foxmail.com; 8 Managing Director Office, Global Business College of Australia, Melbourne, VIC 3000, Australia; siding.luo@eia.edu.au (S.-D.L.); celina.yu@gbca.edu.au (P.Y.); 9 General Manager Office, Edvantage Institute Australia, Melbourne, VIC 3000, Australia; 10 Department of Sports Training, Xi’an Physical Education University, Xi’an 710068, China; huangqian168@126.com

**Keywords:** cognitive–behavioural model, Internet addiction, self-esteem, multiple mediation model, positive and negative affect schedule

## Abstract

The aim of this cross-sectional study was to examine the mediating effects of individual affect and relationship satisfaction on the relationship between self-esteem and Problematic Internet Use (PIU). Affect was measured using the Positive and Negative Affect Schedule (PANAS), relationship satisfaction was assessed using a positive and negative semantic dimension scale, self-esteem was measured using the Rosenberg Self-Esteem Scale, and PIU was measured using the Problematic Internet Use scale with a sample of 507 Chinese university students (Mage = 20.41 years, SD = 2.49). The relationships between the variables were tested using structural equation modelling with a multiple mediation model. The results revealed that negative affect and the negative semantic dimensions of relationship satisfaction mediated the relationship between self-esteem and PIU. The implications of the results and the study’s theoretical contributions are discussed.

## 1. Introduction

The Internet was first introduced to the public as a global computer network in the early 1990s [1,2,3]. The Internet is indispensable for communication, information exchange and sharing, entertainment, socialising, teaching and learning and business development [4,5,6], due to advances in technology and the ubiquity of broadband and mobile phones. However, despite its importance and ease of use, the Internet has the potential to cause harm, as widely acknowledged in academia [7,8,9,10,11,12,13]. Its many advantages may be cancelled out by potential addictive behaviour or problematic use [14].

Problematic Internet Use (PIU) is defined as excessive online activity likely to be associated with significant functional impairment, including compulsive online shopping, gambling, cybersex, and excessive online streaming and social media use that are addictive, impulsive or compulsive elements [15]. Research has shown that problematic internet use is associated with the neglect of important life areas, such as declining educational and work achievement, decreasing sleeping time, reduced quality of meals, and a narrowing range of interests [16,17,18]. An excessive amount of Internet use also has a negative effect on family and partner relations and on communication within the family [19]. Problematic internet use can also cause risk of physical health of individuals [20] or form the basis for criminal activity [21]. Impulsive and compulsive traits can underlie problematic internet behavior [15,22,23,24], during specific internet activities have been associated with psychiatric disorders. For example, online shopping has been linked to depression and hoarding [24]. Grant, et al. [25] noted that addictive behaviour can be conceptualised as substance or non-substance addiction. Problematic Internet Use (PIU) is considered to be a type of non-substance behavioural addiction. Demetrovics, et al. [26] developed a PIU questionnaire, which has become a well-established scale for evaluating addictive Internet behaviour worldwide, including in Africa [27], the Americas [28], Asia [29,30] and Europe [31,32]. The questionnaire was designed to identify the extent of this problem, including whether it is uncontrollable, whether it is markedly distressing and whether it results in social difficulties or hypomania [33]. Factor analysis of the questionnaire of PIU identifies three components: obsession (mental engagement with the Internet), neglect (neglect of everyday activities and essentials needs) and control disorder (difficulties in controlling Internet use) [26]. Other measures have been developed to evaluate specific Internet-based addictive behaviour among adolescents and young adults, including gaming [34,35], gambling [36], social media [37], smartphone use [38] and cybersex [39,40].

Demetrovics, Szeredi and Rozsa [26] developed a PIU questionnaire, which has become a well-established scale for evaluating addictive Internet behaviour worldwide, including in Africa [27], the Americas [28], Asia [29,30] and Europe [31,32]. The questionnaire was designed to identify the extent of this problem, including whether it is uncontrollable, whether it is markedly distressing and whether it results in social difficulties or hypomania [33]. Identifying the mechanisms of PIU can be challenging. The problem has most commonly been approached from the cognitive–behavioural perspective [41,42]. A cognitive behavioural model of internet addiction suggests that low self-esteem is a core aspect of maladaptive cognition of the self, which is the critical factor for problematic internet use [41]. Caplan [42] noted that the ‘virtual interpersonal world might present a serious risk to some individuals’ (p. 573). Other studies have focused on the failure of individuals to control their Internet use and its consequences for social relationships [43,44,45] and academic performance [46,47]. Senol-Durak and Durak [48] claimed that psychosocial well-being, particularly self-esteem, can reduce the likelihood of PIU. Importantly, research has linked self-esteem and PIU in such that low self-esteem is a risk factor for PIU [49] and self-esteem often has identified as a precursor to PIU [50,51,52,53]. A Bangladeshi survey study found that self-esteem is correlated with problematic internet use [52]. There are several studies that also suggest that problematic internet use exerts negative impacts on self-esteem among India adolescents [53], Spanish adolescents [50], and Korean adolescents [51].

Although many studies have found direct relationships between self-esteem and PIU, more research about the internal mechanism is required [54]. Exploring mediating factors might shed light on the relationship between self-esteem and PIU, and advance our understanding of the aetiology of PIU. In this study, we answered the call made by Li, Liu and Dong [54] for further investigation of the internal mechanisms related to self-esteem and PIU. Moreover, we extended the work of Davis [41], Caplan [42] and Senol-Durak and Durak [48] by empirically demonstrating with a sample of university students that individual affect and relationship satisfaction can be significant mediators of PIU.

### 1.1. Self-Esteem and Problematic Internet Use

‘Self-esteem’ refers to one’s subjective evaluation of one’s own worth, and has been extensively examined by psychologists, criminologists and sociologists [55,56,57,58]. According to the risk-buffering hypothesis, favourable individual traits, such as self-esteem, can attenuate the relationship between risk factors and psychological status of individual [59]. Individuals with high levels of self-esteem demonstrated better psychological adjustment such as low negative affect and high positive affect [60], better academic performance [61], lower level of depression and anxiety [62], improved health behaviour and relationship satisfaction [63]. Rosenberg, Schooler and Schoenbach [61], who pioneered the concept of self-esteem and first operationalised its measurement, illustrated its positive effects on academic performance and problems in adolescence. Pyszczynski, Solomon, Greenberg, Arndt and Schimel [62] later suggested that high levels of self-esteem can reduce anxiety and related defensive behaviour, along with recurrent thoughts about death. Further positive impacts of self-esteem have since been identified, such as enhancing social support among in-patients [36], serving as a preventative measure against anxiety during the COVID-19 pandemic [64] and mitigating psychological distress experienced by infertile couples [65]. Promoting self-esteem can help to create positive workplace environments [66] and reduce behaviour detrimental to health [67]. In a cross-cultural study, Diener and Diener [63] identified positive associations between self-esteem and satisfaction with friends, family and life relationships.

Research suggests that self-esteem serves as a preventative factor of PIU [68]. A high level of self-esteem has been found to alleviate the adverse effects of PIU [69,70,71,72,73]. A recent study found that social adjustment and self-esteem had indirect effects, through procrastination, on Internet addiction among college students [74]. Additionally, research has revealed that self-esteem may be indirectly related to academic performance, mental health and problematic social media usage [75,76], and that a combination of self-esteem, a sense of meaning in life and reflection can help reduce the impact of Internet-related addictive behaviour [77,78]. Peng, et al. [79] further suggested that self-esteem was closely associated with Internet addiction, and self-esteem has a mediation effect on the relationship between feeling disconnected from school and Internet addiction, based on a sample of 2758 Chinese adolescents. Therefore, it is important to further investigate the relationship between self-esteem and PIU as well as its underlying mechanism.

### 1.2. Relationship Satisfaction, Individual Affect and Problematic Internet Use

The cognitive–behavioural perspective has been applied in many empirical studies to explore the relationship between relationship satisfaction and PIU. Research found that relationship satisfaction can mediate the effects of PIU [69,80,81,82,83]. Kim and Jun [81] found that the link between Internet use and depression was mediated by relationship satisfaction in a longitudinal study of 4576 older adults. Park, Kang and Kim [69] suggested that children’s relationships with their parents and peers can influence the formers’ PIU. Jeong, Kim, Yum and Hwang [83] argued that PIU can be exacerbated by poor parent–child relationships and low self-esteem. A study of 2758 Chinese adolescents found that school disconnectedness, which refers to students negative connections with their peers, teachers, and community, can exacerbate Internet addiction [79].

Individual affect can also influence Internet-related behaviour. A relationship between negative affect and PIU has also been reported by several empirical studies [84,85,86,87,88,89]. Matthews, Farnsworth and Griffiths [88] revealed that negative mood states can predict students’ involvement in online gambling, a specific category of PIU. Negative mood states were experienced as a consequence of gambling, which in turn facilitated the need for many of the gamblers to modify their mood through play again [90]. Guadagno, Rempala, Murphy and Okdie [89] examined emotional responses to Internet videos and investigated the mechanisms underlying these responses using the Positive and Negative Affect Schedule (PANAS). They found that ‘only content that generates stronger affective responses are likely to spread as a viral video’ (p. 2318). Thus, examining the relationships among self-esteem, individual emotional affect and relationship satisfaction can enhance understanding of the mechanisms of PIU.

### 1.3. Gender Differences of Problematic Internet Use

Gender seems to be an important correlate of PIU, and it is one of the most frequently studies variables in the PIU literature [91]. In the most recent meta-analytical study, it examined 115 independent samples from 34 countries, reporting a global gender difference where men were found to be more prone to PIU symptoms or severity compared to women [92]. Men are about five times more likely to have PIU than women [93]. A study examined the relationship between internet use and gender in 2160 Chinese adolescents and found that males have higher PIU symptoms [94]. Another study also found that men in a sample of 2059 Chinese adolescents scored significantly higher than women in PIU severity [95]. Similarly, males outperformed females in PIU severity in various cultures, including Indian [96], Vietnamese [97], British [98], Romanian [99] and Bengali [100]. In terms of specific categories of internet behaviours, study found that males more often engaged in online role-play games, file downloading, shopping, gambling, visiting pornographic websites, and indiscriminate surfing, whereas the females are more likely to engage in information searching, chatting, e-mailing, and social networking [101].

While the research mentioned above demonstrated such results, many other studies found no significant gender difference in PIU symptoms. A study investigates gender factor in relationship among impulsivity, behavioural inhibition and PIU, and found no gender difference in the severity of the PIU [54]. Similarly, there are many studies that found no gender difference in the severity of PIU in Japanese [102], the United Arab Emirates [103], Portuguese [104] samples. Given the inconsistent findings in the literature, it is important to further investigate the gender factor on PIU.

### 1.4. Purpose of the Study

The purpose of this study was to explore the relationships among self-esteem, individual affect, relationship satisfaction and PIU, and to test the mediating roles of both individual affect and relationship satisfaction on the link between self-esteem and PIU. Based on the results of previous studies, we proposed and tested the following three hypotheses regarding the relationships among self-esteem, individual affect and relationship satisfaction and their influence on PIU. We also tested whether the mediation effects differed by gender through the multigroup mediation model.

**Hypothesis** **1** **(H1).** *Self-esteem would be negatively related to problematic internet use*.

**Hypothesis** **2** **(H2).** *Negative affect would mediate the effect of self-esteem on problematic internet use*.

**Hypothesis** **3** **(H3).** *Relationship dissatisfaction would mediate the relationships between self-esteem and problematic internet use*.

**Hypothesis** **4** **(H4).** *There is a gender difference on relationships among self-esteem, individual affect, relationship satisfaction, and PIU*.

## 2. Methods

### 2.1. Participants

We conducted our cross-sectional research in April and May 2019 as part of a broader study related to quality of life and Internet use among students at a university in Guangzhou, China. We recruited 507 undergraduate students (Mage = 20.41 years, SD = 2.49, 14.5% male) through the university’s intranet system using a self-report smartphone-based application. The recruited sample reflected the demographic profile of the university population. The participants were asked to provide their informed consent before voluntarily participating in the study. The research process was conducted in accordance with the relevant rules and regulations set out in the Statistics Law of the People’s Republic of China and the Declaration of Helsinki, acknowledging their revisions.

### 2.2. Measures

The PIU questionnaire [26] measured the three latent factors of obsession, neglect and control disorder, with six items per subscale. The participants were asked to rate each item on a 5-point Likert-type scale ranging from 1 = never to 5 = always. The questionnaire has been validated in a Chinese context [30] and has been frequently used to analyse PIU among Chinese adolescents [105]. The scale in this study had good reliability, with a Cronbach’s α of 0.862.

PANAS [106] includes both positive and negative affect factors, with 10 items per factor. Items are measured on a 5-point Likert-type scale. The overall reliability of PANAS is 0.846. In this study, the Cronbach’s α values for the subscales of positive affect (PA) and negative affect (NA) were 0.802 and 0.884, respectively, in line with those in other PANAS studies in the context of Chinese society [65,107,108].

The positive and negative semantic dimensions of relationship satisfaction were measured using a relationship satisfaction scale (PN-SMD) [109]. The participants were asked to rate the 14 items (7 positive and 7 negative) on an 8-point Likert-type scale from 0 (not at all) to 7 (always). The PN-SMD has been used to examine deviant Internet behaviour among Chinese adolescents [110] and behaviour manifesting alcohol addiction in couples [111]. The Cronbach’s α values for the PN-SMD scale as a whole, the positive semantic dimension (P-SMD) subscale and the negative semantic dimension (N-SMD) subscale were 0.764, 0.905 and 0.922, respectively.

Self-esteem was measured using the 10-item Rosenberg Self-Esteem Scale (RSES) [61]. This is a 5-point Likert-type scale in which 0 = strongly disagree and 4 = strongly agree. The Chinese version of the RSES has been validated [112,113,114] and the scale had good reliability in the present study, with Cronbach’s α = 0.763.

### 2.3. Procedure

We first conducted confirmatory factor analysis (CFA) to ensure the validity of the five scales. We treated the response data as continuous for all of the scales and conducted our CFA with a maximum likelihood estimator, as the data were normally distributed, i.e., skewness < 2 and kurtosis < 7 [115,116].

The latent variable models were assessed using multiple fit indices. Our criteria were that (1) the Tucker–Lewis index (TLI) and the comparative fit index (CFI) should both be equal to or greater than 0.9 [117,118,119]; and (2) the root mean square error of approximation (RMSEA) and standardized root mean square residuals (SRMR) should both be no more than 0.08 [120].

The results of the validity testing are given in Table 1. The good model fit indicated that the scales we used had high construct validity.

The correlations between the variables were consistent with our assumptions (Table 2). The preliminary correlation results also supported the significant relationships observed between the variables for the male and female participants and the magnitudes of these relationships (Table 3). We then further analysed the mediation effects among the variables through our multigroup mediation model.

## 3. Results

### 3.1. Mediation Model

**Figure 1:**
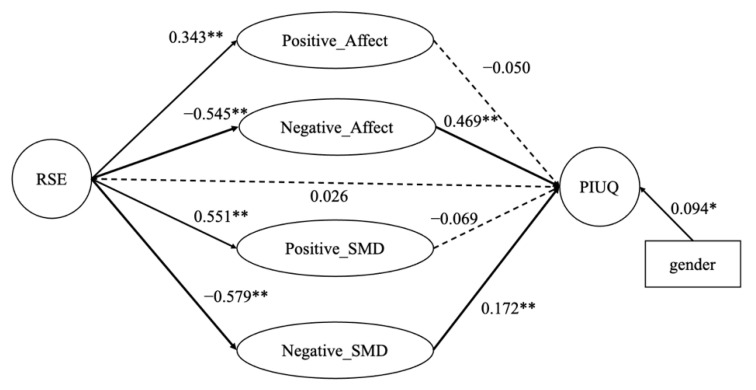
Multiple mediation model. Note: * *p* < 0.05, ** *p* < 0.01. The solid lines represent significant path coefficients, and the bold solid lines indicate the mediation effects. The dotted lines represent non-significant path coefficients.

We developed a multiple mediation model [121] of the PIU questionnaire to simultaneously test the indirect effects of the PANAS and PN-SMD measures while controlling for gender. The data analyses were conducted using Mplus 8.4 [122]. The model fit was good (CFI = 0.930, TLI = 0.917, SRMR = 0.075, RMSEA = 0.064, 90% CI of RMSEA = [0.058, 0.070]). Table 4 and Figure 1 give the standardised coefficients of the multiple mediation model, indicating that the relationship between RSE and PIU was mediated by negative affect (β = −0.256, *p* < 0.001,95% CI = (−0.460, −0.221)) and negative SMD (β = −0.100, *p* = 0.004, 95% CI = (−0.235, −0.029)).

Before implementing the multigroup mediation model, we tested the measurement invariance of the model’s four scales between the male and female participants. First, we tested the configural invariance of all of the scales and found that the structures of the four scales were the same for the male and female samples, demonstrating a good model fit (Table 5, Table 6, Table 7 and Table 8). We then constrained the loadings to be the same for both genders (metric invariance) and assessed whether the model’s fit was significantly different from the fit of the configural invariance model (ΔCFI should be less than 0.01) [123]. Our results showed that all of the scales satisfied the requirement of metric invariance. We then applied scalar invariance models in which the intercepts of the observed variables were set as equal for the different groups. The results showed that the fit of the scalar invariance models was as good as that of the metric invariance models (ΔCFI < 0.01). Thus, measurement invariance between the male and female participants was demonstrated for all of the models.

### 3.2. Multigroup Mediation Model

We tested whether the mediation effects differed by gender through the multigroup mediation model. Table 9 gives the mediation effects for male and female groups. We used the Wald test to identify any differences in the mediation effects between the two groups [123]. The mediation effect of negative SMD was found to differ significantly (W_dif4_ = −0.249, *p* = 0.039). For the male participants, it mediated the relationship between RSE and PIUQ (β_4male_ = −0.252, *p* = 0.004, 95% CI = [−0.808, 0.059]), but this effect was not significant for the female participants (β_4female_ = −0.034, *p* = 0.396, 95% CI = [−0.130, 0.052]). We found no significant differences in the other mediation effects (W_dif1_ = 0.010, *p* = 0.781; W_dif2_ = 0.079, *p* = 0.521; W_dif3 = 0.004, *p* = 0.956). The mediation effect of negative SMD was significant in the female sample (β_2female_ = −0.255, *p* < 0.001, 95% CI = [−0.345, −0.174]) but not in the male sample (β_2male_ = −0.167, *p* = 0.097, 95% CI = (−0.838, 0.086)). However, this difference was not significant.

## 4. Discussion

We examined the relationships among self-esteem, affect, relationship satisfaction and PIU. Echoing previous studies, the findings of our bivariate and multivariate analyses suggested that self-esteem was significantly negatively related to problematic internet use [63,86]. Hypothesis 1 was therefore supported. The results reported in Table 2 found a non-significant relationship between PA and PIU, with r = −0.003, whereas P-SMD showed a significant negative relationship (r = −0.241, *p* < 0.01) with PIU. The observed relationships and their magnitudes remained similar after the data were divided by gender (Table 3). The multiple mediation model further confirmed these relationships (Figure 1 and Table 4). Neither positive affect nor relationship satisfaction was significantly related to PIU, again supporting previous findings [82,124].

Our main contribution lies in our illustration of the mediating roles of both negative individual affect and relationship dissatisfaction in the association between self-esteem and PIU. Using a multiple mediation model, we found that negative scores for affect (β = −0.256, *p* < 0.001) and relationship dissatisfaction (β = −0.100, *p* = 0.004) mediated the relationship between self-esteem and PIU (Figure 1 and Table 4), supporting Hypothesis 2 and 3. However, although both positive affect and relationship satisfaction were related to PIU, neither mediated the relationship between self-esteem and PIU. Our results also suggest a gender difference in terms of PIU (Table 9), which supports Hypothesis 4. Our findings therefore provide empirical evidence in line with the cognitive–behavioural perspective [41,42,125]. In short, the current study provides empirical evidence to support the claim that ‘social isolation and lack of self-esteem might play in the PIU process’ [42] (p. 569). As such, the findings further clarified the internal mechanisms related self-esteem and PIU which reported in the existing literature [54], by illustrating that negative affect and poor relationships mediate PIU.

Our study has several limitations. First, the sample was restricted to young adults, but psychological mechanisms and behavioural patterns related to Internet use may vary due to demographic and age differences. The cross-age stability and applicability of our conclusions could be examined in future research using samples from various age groups. Second, our sample is taken from a school in southern China. Due to China’s vast territory and large population, there are also differences in population distribution between the north and the south. There may be doubts whether our sampling can represent all college students in China. Moreover, the ratio of males to females in the sample of this study does not necessarily represent the national college students. Future research can take research samples from all provinces in China, which can improve the generalizability of research conclusions. Third, our main approaches were a self-report measure and a cross-sectional design, preventing us from establishing causality among the variables. In future research, a longitudinal design could be used, with lab experiments conducted to examine causality. Fourth, our proposed multiple mediation model indicated that negative affect and relationship dissatisfaction only partially accounted for the effect of self-esteem on PIU. Therefore, other factors may be involved in this effect. Other psychological variables should therefore be explored in future studies.

Despite these limitations, we confirmed that negative affect and relationship dissatisfaction mediated the relationship between self-esteem and PIU. This finding has significant practical implications for mitigating PIU among young adults. Drawing on the cognitive–behavioural model [41,42], our results indicated that negative emotions and interpersonal dissatisfaction are closely related to PIU. Thus, practical and effective interventions and training could help young adults alleviate their PIU, by enabling them to regulate their emotions and build satisfactory interpersonal relationships. We also found interpersonal dissatisfaction to be an important potential cause of PIU. Schools, teachers and parents should therefore be aware that helping young adults develop satisfactory interpersonal relationships is extremely important. Relevant interventions could include the teaching of skills for and practical methods of developing such relationships. Education departments, schools and parents should offer advice on and training in emotional management, maintaining interpersonal relationships and ensuring healthy communication. They should also organise activities to enhance young adults’ social relations and interpersonal satisfaction. If young adults have a healthy emotional outlook, are able to regulate their emotions, and have good interpersonal communication skills and sound interpersonal relationships, their PIU is likely to diminish.

## 5. Conclusions

We investigated the relationships among self-esteem, individual affect, relationship satisfaction, and PIU. Mediation analysis indicated that negative affect and relationship dissatisfaction mediated the relationship between self-esteem and PIU. Interestingly, neither positive affect nor relationship satisfaction mediated the relationship between self-esteem and PIU. The present study identifies a mechanistic pathway (i.e., via negative affect, relationship dissatisfaction) through which we can better understand the critical process by which self-esteem buffers PIU. Therefore, PIU intervention programs in young adults should focus more on relieving relationship dissatisfaction and negative affect than on improving positive affect and relationship satisfaction.

## Figures and Tables

**Table 1:** Confirmatory factor analysis.

Scales	CFI	TLI	SRMR	RMSEA	90%CI of RMSEA
PIUQ	0.928	0.902	0.043	0.054	(0.046, 0.062)
PANAS	0.916	0.903	0.048	0.058	(0.052, 0.065)
PN-SMD	0.918	0.902	0.042	0.100	(0.091, 0.109)
RSE	0.942	0.913	0.053	0.066	(0.051, 0.081)

**Table 2:** Correlation matrix of all variables (*n* = 507).

	Mean	SD	1	2	3	4	5
1. PIUQ	47.945	9.828					
2. Positive_Affect	29.892	5.833	−0.003				
3. Negative_Affect	24.022	7.267	0.471 **	0.191 **			
4. Positive_SMD	32.616	7.655	−0.241 **	0.377 **	−0.241 **		
5. Negative_SMD	20.448	8.376	0.369 **	−0.131 **	0.451 **	−0.502 **	
6. RSE	26.667	3.935	−0.296 **	0.275 **	−0.454 **	0.420 **	−0.443 **

Note: * *p* < 0.05, ** *p* < 0.01.

**Table 3:** Correlation matrix of all variables (split by gender).

	Male (*n* = 73)					Female (*n* =434)				
	1	2	3	4	5	1	2	3	4	5
1. PIUQ										
2. Positive_Affect	0.015					−0.007				
3. Negative_Affect	0.526 **	0.205				0.465 **	0.187 **			
4. Positive_SMD	−0.352 **	0.380 **	−0.300 **			−0.215 **	0.377 **	−0.226 **		
5. Negative_SMD	0.605 **	−0.016	0.566 **	−0.405 **		0.307 **	−0.156 **	0.424 **	−0.525 **	
6. RSE	−0.218	0.159	−0.418 **	0.277 *	−0.300 **	−0.321 **	0.298 **	−0.459 **	0.449 **	−0.475 **

Note: * *p* < 0.05, ** *p* < 0.01.

**Table 4:** Standardised results of mediation effects.

Path	β	p	95% CI ^1^
RSE-Positive Affect-PIUQ	−0.017	0.396	(−0.095, 0.034)
RSE-Negative Affect-PIUQ	**−0.256**	**0.000**	**(−0.460, −0.222)**
RSE-Positive SMD-PIUQ	−0.038	0.258	(−0.139, 0.051)
RSE-Negative SMD-PIUQ	**−0.100**	**0.004**	**(−0.235, −0.029)**

^1^ 95% confidence interval of bootstrapping procedure with 1000 bootstrap draws. Note: bold = significant mediation effect.

**Table 5:** Measurement invariance of RSE.

Model	CFI	TLI	SRMR	RMSEA	90%CI of RMSEA	ΔCFI	Pass?
Configural Invariance	0.938	0.903	0.055	0.070	(0.054, 0.086)		Yes
Metric Invariance	0.940	0.919	0.059	0.064	(0.049, 0.079)	0.002	Yes
Scalar Invariance	0.941	0.930	0.061	0.060	(0.045, 0.075)	0.001	Yes

**Table 6:** Measurement Invariance of PIUQ.

Model	CFI	TLI	SRMR	RMSEA	90%CI of RMSEA	ΔCFI	Pass?
Configural Invariance	1.000	1.000	0.000	0.000	(0.000, 0.000)		Yes
Metric Invariance	1.000	1.000	0.014	0.000	(0.000, 0.069)	0.000	Yes
Scalar Invariance	0.995	0.992	0.038	0.061	(0.000, 0.125)	0.005	Yes

Note: Due to the complexity of the model structure of PIUQ scale and the small sample size of the male group, the multigroup model did not converge. Therefore, we packaged the 18 observed variables of PIUQ scale and then conducted multigroup model for this scale.

**Table 7:** Measurement invariance of PANAS.

Model	CFI	TLI	SRMR	RMSEA	90%CI of RMSEA	ΔCFI	Pass?
Configural Invariance	0.907	0.891	0.058	0.063	(0.056, 0.070)		Yes
Metric Invariance	0.903	0.893	0.065	0.062	(0.055, 0.069)	0.004	Yes
Scalar Invariance	0.899	0.894	0.065	0.062	(0.055, 0.068)	0.004	Yes

**Table 8:** Measurement Invariance of PN-SMD.

Model	CFI	TLI	SRMR	RMSEA	90%CI of RMSEA	ΔCFI	Pass?
Configural Invariance	0.925	0.910	0.045	0.096	(0.087, 0.106)		Yes
Metric Invariance	0.925	0.917	0.048	0.093	(0.084, 0.102)	0.000	Yes
Scalar Invariance	0.920	0.920	0.048	0.090	(0.082, 0.099)	0.005	Yes

**Table 9:** Standardised results of mediation effects (split by gender).

	Male			Female		
Path	β	p	95% CI ^1^	β	p	95% CI ^1^
1. RSE-Positive Affect-PIUQ	−0.007	0.769	(−0.170, 0.073)	−0.018	0.460	(−0.070, 0.037)
2. RSE-Negative Affect-PIUQ	−0.167	0.097	(−0.838, 0.086)	**−0.255**	**0.000**	**(−0.345, −0.174)**
3. RSE-Positive SMD-PIUQ	−0.032	0.553	(−0.286, 0.071)	−0.039	0.331	(−0.123, 0.048)
4. RSE-Negative SMD-PIUQ	**−0.252**	**0.004**	**(−0.808, −0.059)**	−0.034	0.396	(−0.130, 0.052)

^1^ 95% confidence interval of bootstrapping procedure with 1000 bootstrap draws. Note: bold = significant mediation effect.

## Data Availability

The datasets used and analyzed during the current study are available from the correspondent author on reasonable request.
